# High-Throughput Screening of a *Corynebacterium glutamicum* Mutant Library on Genomic and Metabolic Level

**DOI:** 10.1371/journal.pone.0086799

**Published:** 2014-02-04

**Authors:** Lorenz C. Reimer, Jana Spura, Kerstin Schmidt-Hohagen, Dietmar Schomburg

**Affiliations:** Department of Bioinformatics and Biochemistry, Technische Universität Braunschweig, Braunschweig, Germany; Imperial College London, United Kingdom

## Abstract

Due to impressive achievements in genomic research, the number of genome sequences has risen quickly, followed by an increasing number of genes with unknown or hypothetical function. This strongly calls for development of high-throughput methods in the fields of transcriptomics, proteomics and metabolomics. Of these platforms, metabolic profiling has the strongest correlation with the phenotype. We previously published a high-throughput metabolic profiling method for *C. glutamicum* as well as the automatic GC/MS processing software MetaboliteDetector. Here, we added a high-throughput transposon insertion determination for our *C. glutamicum* mutant library. The combination of these methods allows the parallel analysis of genotype/phenotype correlations for a large number of mutants. In a pilot project we analyzed the insertion points of 722 transposon mutants and found that 36% of the affected genes have unknown functions. This underlines the need for further information gathered by high-throughput techniques. We therefore measured the metabolic profiles of 258 randomly chosen mutants. The MetaboliteDetector software processed this large amount of GC/MS data within a few hours with a low relative error of 11.5% for technical replicates. Pairwise correlation analysis of metabolites over all genotypes showed dependencies of known and unknown metabolites. For a first insight into this large data set, a screening for interesting mutants was done by a pattern search, focusing on mutants with changes in specific pathways. We show that our transposon mutant library is not biased with respect to insertion points. A comparison of the results for specific mutants with previously published metabolic results on a deletion mutant of the same gene confirmed the concept of high-throughput metabolic profiling. Altogether the described method could be applied to whole mutant libraries and thereby help to gain comprehensive information about genes with unknown, hypothetical and known functions.

## Introduction

The technological progress in genomic research led to a dramatic increase of knowledge of whole genome sequences, opening the demand of understanding the complex and dynamic processes of gene expression, proteomics and metabolic pathways. While a rapid development in multiparallel analytical methods for transcriptomics and proteomics has already taken place [1] the field of metabolomics still lacks appropriate techniques. Only in recent years first metabolic high-throughput methods were published [2]–[5].

Compared to the other Omics-platforms, metabolomics closely reflects cell activity at the functional level and therefore is often directly correlated with the cellular phenotype. Observed changes in transcriptome and proteome level do not always correspond to phenotypic alterations [6]. Even the interpretation of metabolic data is often not straightforward. Due to the convoluted state of cell metabolism, where many metabolites are involved in different pathways, it is difficult and sometimes even impossible to establish a direct link between genes and metabolites [6].

The Gram-positive soil bacterium *Corynebacterium glutamicum* is widely used in the industrial production of amino acids such as L-glutamate and L-lysine [7]. Two independent groups published the genome of *C. glutamicum,* containing approximately 3000 genes [8], [9]. Applying homology studies, functions could be assigned to around 83% of the protein-coding genes, many of them putative or even highly speculative [8].

Nowadays large transposon mutant libraries are available for defined organisms, applying random transposon mutation [10]–[12]. For example, *C. glutamicum* was used to generate over 10,000 [13] or even 18,000 [14] mutants. Moreover, Suzuki et al. [14] added a high-throughput transposon insertion location method, working with a thermal asymmetric interlaced PCR (TAIL-PCR) [15] to amplify transposon border regions. Using the sequenced TAIL-PCR products with BLAST, 18,000 mutants were identified. Such a library of identified mutants offers a valuable source for research in systems biology.

We [3] developed a high-throughput method for the analysis of metabolic profiles of *C. glutamicum* mutants and showed, that it is possible to measure 72 samples per day, assuring high sensitivity as well as good reproducibility of the analysis. Nevertheless, our method revealed two small bottlenecks in throughput: insertion site determination and data processing. While the measurement of hundreds of mutants could be carried out within days, processing of the corresponding GC/MS data took weeks because of the absence of appropriate software. Secondly, the determination of the insertion sites was missing a high-throughput method and therefore only single mutants were identified.

In recent years, several automatic GC/MS processing software packages have been developed amongst others: TagFinder [16], MetaboliteDetector [17], MetAlign [18], Mzmine 2 [19], which all reduce the processing time significantly. In our study we used a further developed version of our in-house developed software MetaboliteDetector to process 861 samples automatically, comprising 774 mutant, 45 wild type and 42 quality standard samples within a few hours. Together with the here-described adapted high-throughput insertion point determination for the *C. glutamicum* transposon mutant library, we were able to study changes in the metabolic phenotype and combine these with data of the genomic background for every single mutant of this random transposon mutant library.

## Results

### High-throughput Insertion Point Determination

An insertion point determination was established using the TAIL-PCR method [20] for our in-house *C. glutamicum* transposon mutant library. This transposon mutant library was created by the aid of the vector pAT6100 [13] and the restriction deficient wild type derivative *C. glutamicum* Res167. As the success-rate of the TAIL-PCR is dependent on the binding of the degenerated primer close to the transposon resulting from the pAT6100 vector in the genome, different degenerated primer pairs were used in parallel to increase efficiency. The adapted procedure was used for the screening of 1152 mutants. Only mutants that resulted in an adequate amplification regarding quality and length of the PCR product were used for sequencing. For 87% of the submitted DNA fragments the length of the sequence was sufficient to successfully identify the mutant via a BLAST search, leading to the accurate insertion point determination of 722 mutants in total.

**Figure 1:**
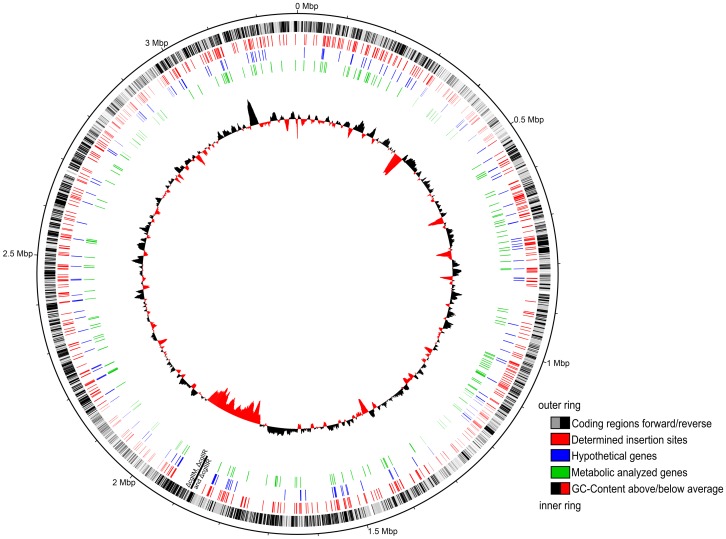
722 transposon insertion points plotted onto the genome of C. glutamicum ATCC 13032 (Genbank Acc. No. NC006958). The plot was generated with DNAPLOTTER 1.4. The circles show (from outward to inward): (I) coding regions (forward: grey, reverse: black), (II) overview of all determined insertion sites (red), (III) identified genes with hypothetical function (blue), (IV) insertion sites of mutants that were analyzed within the high-throughput metabolic profiling (green), (V) GC content (black: above average, red: below average) and across circle (II) to (IV) the deleted genes in the restriction deficient wild type modification Res167 (black: ΔcglIM, ΔcglIR and ΔcglIIR).

As described before by Tauch et al. (2002) [21], the vector pAT6100 with its transposable element IS6100 is randomly integrating into the genome of *C. glutamicum* and has no site-preference. To prove this, we plotted all 722 determined insertion points onto the genome of *C. glutamicum* ATCC 13032 (figure 1). As expected, the distribution of the transposon insertion sites over the whole genome was random. Table 1 shows a summary of the identified gene loci. Over 85% of the transposons hit a gene, strikingly close to the coding density of 87% in *C. glutamicum*, which therefore indicates a representative distribution of mutations in the genome. For 36% of the here described mutated genes no or only a hypothetical function was found. This is more, than the 17% reported by Kalinowski et al. (2003) [8]. All 611 affected genes were further analyzed by an alignment generated with our in-house developed software tool EnzymeDetector [22]. EnzymeDetector automatically compares and evaluates assigned enzyme functions from the main annotation databases and supplements them with its own function prediction. With the aid of EnzymeDetector another 20 hypothetical genes were identified, e.g. the gene *NCgl0528* encoding the pyruvate water dikinase (EC 2.7.9.2), which is annotated as hypothetical protein in the NCBI database. Similarly, we found additional information for many other genes. In total, only 67 of the mutated genes were already investigated in *C. glutamicum* and discussed in publications. This underlines the need for high-throughput techniques to analyze not only the function of single genes, but the functions of large sets of genes.

**Table 1:** Overview of the insertion point determination.

	Sum	Coverage [%]
Located insertion points of transposons	722	
Transposon hits a gene	611	85^1^
Genes within an operon	334	55^2^
Genes with unknown orhypothetical function	222	36^2^
Genes investigated in *C.glutamicum*	67	11^2^

1 data refer to overall located transposons;

2 data refer to transposons that hit a gene.

### High-throughput Metabolic Profiling

**Figure 2:**
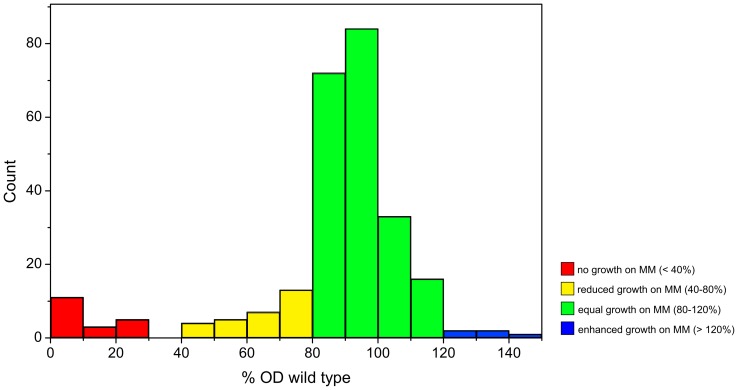
Histogram displaying the relative optical density (% OD) compared to the C. glutamicum wild type. Measurements were done after 7 hours incubation in glucose minimal medium (MM). Mutants can be divided into 4 groups: no or only minimal growth on MM (red), reduced growth (yellow), equal growth (green) and enhanced growth (blue) compared to the wild type.

In a pilot project, comprising the metabolome analysis of 258 randomly chosen mutants (table S1) of the *C. glutamicum* transposon mutant library, we used our previously published method for metabolic profiling [3]. An overview of the relative growth of all investigated mutants on minimal medium compared to the wild type is shown in figure 2. The mutants can be divided into four groups. A first group including 22 mutants showed no or only minimal growth. While these mutants were able to grow on complex medium so that the insertion point of the transposon could be determined, the analysis of the metabolic profile on minimal medium was impossible because of low biomass yields. Still, these mutants are interesting, since the mutation damages essential parts for growth on minimal medium with glucose as the sole carbon source. Therefore it is not surprising to find mutations in genes encoding for enzymes involved in glycolysis (*NCgl1526* encoding the glyceraldehyde-3-phosphate dehydrogenase, EC 1.2.1.12), the pentose phosphate pathway (*NCgl1536* encoding the ribulose-phosphate 3-epimerase, EC 5.1.3.1) or the valine, leucine and isoleucine pathway (*NCgl1219* encoding the dihydroxy-acid dehydratase, EC 4.2.1.9 and *NCgl0245* encoding the 2-isopropylmalate synthase, EC 2.3.3.13). Also an intergenic mutation (mutant P21E12), that did not hit a gene directly was found within this group. A closer look revealed that this mutation is located 18 bases upstream of the gene *NCgl0935*, encoding the phosphopyruvate hydratase (EC 4.2.1.11). This might lead to a damage of the promoter region causing a decrease in gene expression of *NCgl0935*. It is noteworthy, that within this small group five of the affected genes have already been investigated before including some of the already mentioned enzymes ([23], [24], [25], [26], [27]). Another 29 mutants form the second group that showed reduced growth (40–80%) compared to the wild type. This group comprises the most interesting targets for metabolic profiling, since the mutations affect growth noticeably, but the growth is still sufficient for metabolome analysis. By far the largest group, with 204 mutants, contains the mutants with growth similar to the wild type (80–120%). These mutants comprise a mixture of silent mutations and mutations with measurable influence on the organism, still allowing comparable growth to the wild type on minimal medium. The smallest group of mutants includes five mutants that showed enhanced growth compared to the wild type (>120%).

For the analysis of the GC/MS samples, we used our in-house software MetaboliteDetector [17] in the version 2.0.7. MetaboliteDetector is a device-independent GC/MS data processing software, that automatically detects, identifies and quantifies metabolites in either a targeted or non-targeted approach. Metabolite identification was performed with a combined library, achieved by a merge of our in-house library with the Golm Metabolome Database [28]. This combined library comprises more than 2500 substances, that can be divided into 1522 metabolite derivatives and 1053 unidentified substances. In a non-targeted mode, MetaboliteDetector detected up to 292 substances with biological response in the *C. glutamicum* samples. In total, we found 186 distinct substances in all experiments including unidentified substances. The overall relative error, determined by taking the mean of the relative error of all identified metabolites in the quality standards, resulted in a value of 11.5%, which underlines the good reproducibility and stability of the method. A list of all reproducibly quantified metabolites in the wild type is shown in the supplemental material (table S2).

### Pairwise Correlation Analysis

In contrast to mRNA and proteins, metabolites are not newly synthesized but are formed by transformation of precursor metabolites [29]. Because of this close connection to other metabolites and pathways they are highly dependent upon each other, which can be determined by correlation analysis, principal component analysis or hierarchical cluster analysis. Steuer et al. [30] analyzed pairwise correlations of metabolites within biological replicates of potato plants and found that even changes within the metabolites of biological replicates with identical genotype and grown under the same conditions were sufficient to draw specific patterns in the pairwise correlation of the metabolites. This pattern can be seen as a unique fingerprint.

**Figure 3:**
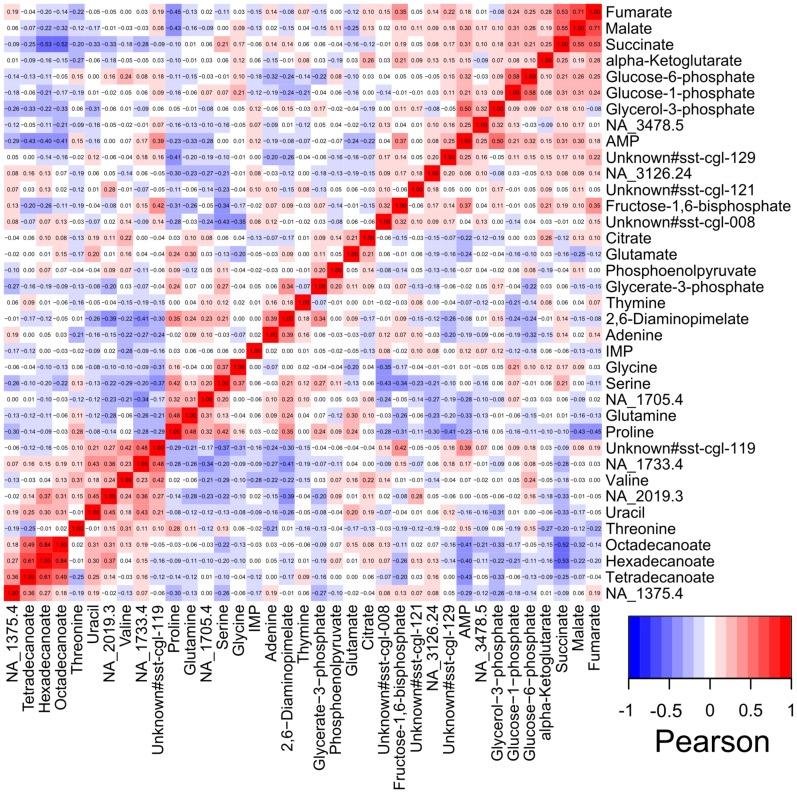
Heatmap of pairwise correlation values of 37 metabolites. The Pearson correlation coefficients were calculated for log2 transformed ratios of the median values of C. glutamicum mutant and wild type. For a better overview only metabolites with the highest reproducibility (>95%) were displayed.

Unlike Steuer et al., we analyzed the pairwise correlation of metabolites not within one genotype, but over many different genotypes. Due to the high number of genetic perturbations, the dependencies of metabolites in the metabolic response of *C. glutamicum* should become visible. Before correlating the ratios the values were log2 transformed. Thereby a robust image of the dependencies of the metabolites should be generated. As can be seen in figure 3 the metabolites show correlations in the range of −0.53…0.84. Since the pairwise correlation was done with a high number of different genotypes, which also provide unspecific changes in their metabolic profile, the correlations are quite low. Nevertheless, tendencies in the correlation of metabolites can be seen. The metabolites of the TCA cycle, especially fumarate, malate and succinate show a high correlation. Also metabolites associated with glutamate like glutamine and proline show high correlations as well as the fatty acids octadecanoate, hexadecanoate and tetradecanoate. In addition, high negative correlations were found for AMP and succinate to some fatty acids and for the non identified metabolite unknown#sst-cgl-008 to serine and glycine.

**Figure 4:**
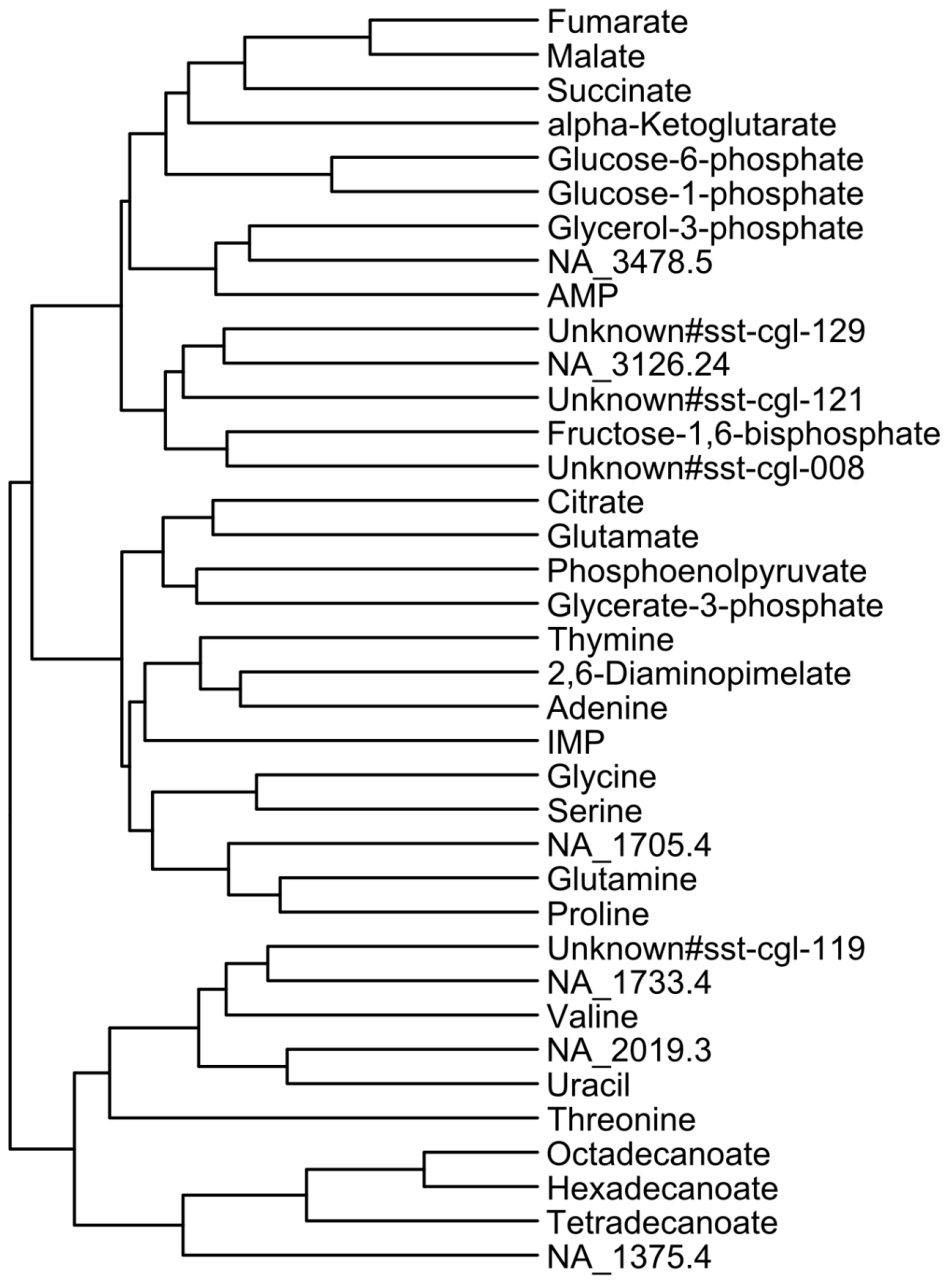
Dendrogram based on the correlation values in figure 3. For the calculation of the distance the euclidean distance was used.

The matrix of correlation values in figure 3 provides detailed information but for a better overview of the relationships between the metabolites, the complexity has been reduced by a dendrogram shown in figure 4. This kind of presentation of the pairwise metabolite correlation shows expected connections and reflects already known structures in the metabolism. The metabolites of the TCA cycle fumarate, malate, succinate and α-ketoglutarate, with exception of citrate, form one group. Similarly, metabolites of the glycolysis like phosphoenolpyruvate and glycerate-3-phosphate are arranged in another group, while fructose-1,6-bisphosphate is not associated with them. Metabolites associated with glutamate like glutamine and proline form another group while glutamate itself can be found in a group together with citrate. Very interesting is the connection of so far non identified metabolites to known metabolites. The metabolite NA_1705.4 shows a correlation to the glutamate associated metabolites glutamine and proline. Another non identified metabolite NA_1375.4 is associated with the fatty acids (figure 4).

### Screening for Pattern in Specific Pathways

As shown in the pairwise correlation analysis, metabolites are highly dependent on each other. Consequently, mutations often do not affect only single metabolites but pairs or even whole sets of closely related metabolites. We used information gathered by the pairwise correlation of metabolites to search for patterns in the metabolic profiles. The data set was screened for accumulation of significant changes in specific pathways.

**Figure 5:**
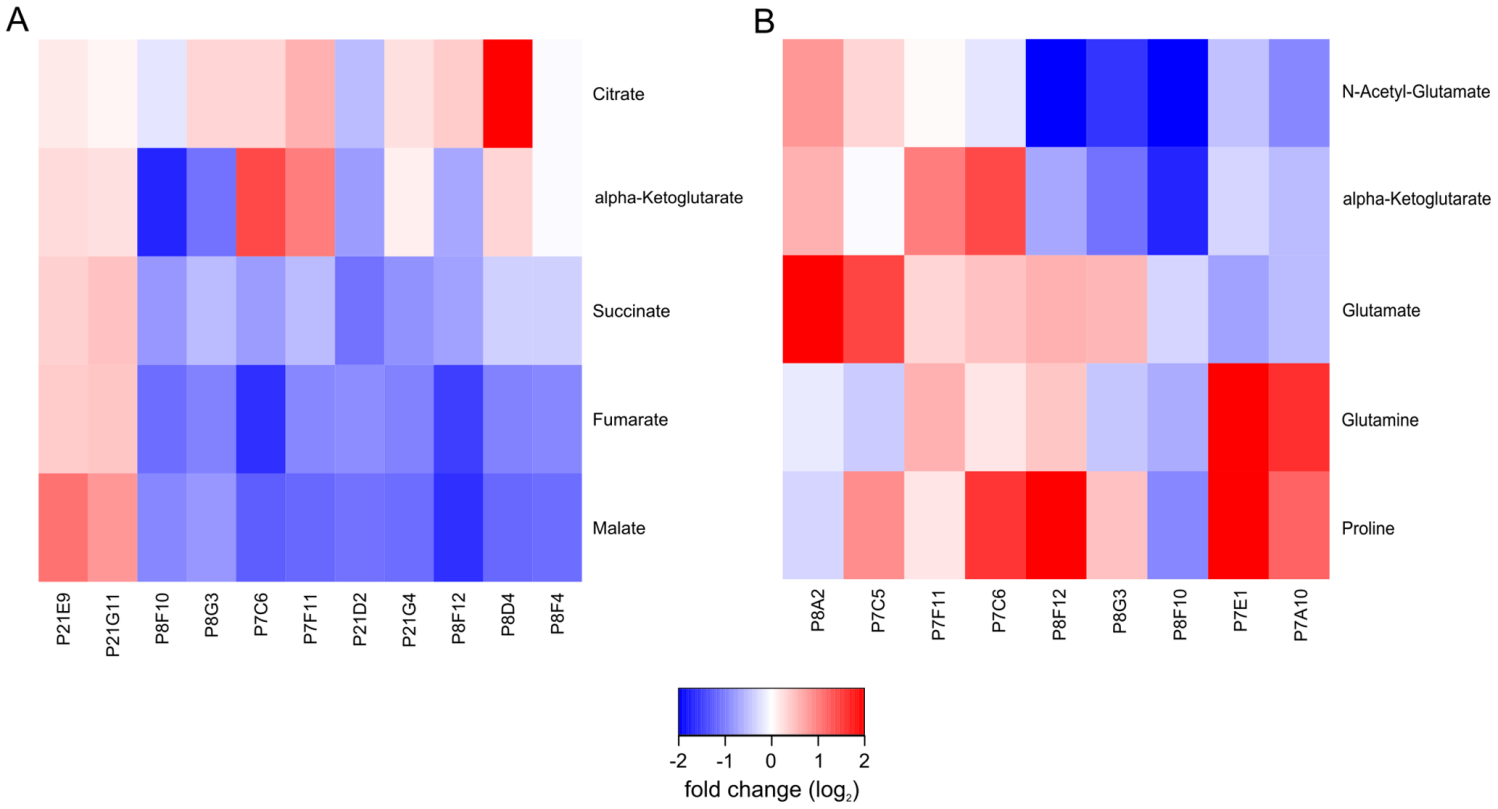
Heatmap showing log2 transformed ratios (mutant/wild type) of metabolites in selected pathways. Only C. glutamicum mutants with significant changes (p<0.01 Bonferroni corrected) in at least 2 of the selected metabolites were analyzed. **A** Ratios of metabolites in the TCA cycle. **B** Ratios of metabolites associated with glutamate.

At first the TCA cycle was analyzed. Figure 5A shows a heatmap representing the peak area ratios of TCA cycle intermediates between 11 selected mutants and the wild type. Mutants were selected for this plot when at least two metabolites of the TCA cycle showed significant changes compared to the wild type (p-value 0.01, adjusted by Bonferroni correction). As expected, fumarate, malate, and succinate are co-regulated, while the early TCA cycle metabolites citrate and α-ketoglutarate seem to be regulated independently from the ones mentioned above. Fumarate, malate and succinate also formed a group in the pairwise correlation of metabolites over all mutants (figure 4). While citrate was separated from the other metabolites of the TCA cycle, α-ketoglutarate was associated with the group of fumarate, malate and succinate in the pairwise correlation analysis.

A similar approach was applied to investigate selected metabolites associated with the glutamate metabolism (figure 5B). Unlike the results for the TCA cycle intermediates, the ratios of the glutamate related metabolites did not show a homogeneous picture. Interestingly, the two mutants P7E1 and P7A10 showed a similar pattern regarding metabolite ratios. While the levels of proline and glutamine were increased, the levels of glutamate, α-ketoglutarate and N-acetyl-glutamate were slightly decreased. In the mutant P7A10 the gene *glnE* (*NCgl2147)* is affected, which is encoding for the glutamate-ammonia-ligase adenylyltransferase (EC 2.7.7.42) that is involved in the regulation of ammonium uptake. In the mutant P7E1 the insertion point of the transposon is still unknown.

A list of the analyzed mutants included in the figures 5A and 5B is shown in table 2. Five mutants showed significant changes in the TCA cycle, as well as in glutamate associated metabolites, whereas the others were either involved in the TCA cycle or the glutamate metabolism. In the following section several mutants that showed an interesting metabolic response compared to the wild type are investigated in detail.

**Table 2:** List of *C. glutamicum* mutants with changes in the TCA cycle or in glutamate associated metabolites (GLU), or in both (bold).

ID	Position	Locus	Gene description	Operon	EC	Pearson	% OD	TCA	GLU
P7A10	2361816	NCgl2147	Glutamate-ammonia-ligase adenylyltransferase	–	2.7.7.42	0.97	67		+
P7C5	2238004	NCgl2038	Hypothetical protein	–	–	0.96	107		+
**P7C6**	**Not identified**					**0.92**	**50**	**+**	**+**
P7E1	Not identified					0.92	91		+
**P7F11**	**179131**	**NCgl0163**	**Major facilitator superfamily permease**	**+**	**–**	**0.97**	**83**	**+**	**+**
P8A2	368514	NCgl0340	Nucleoside-diphosphate sugar epimerase	+	–	0.96	89		+
P8D4	Not identified					0.93	42	+	
P8F4	1042752	NCgl0944	Major facilitator superfamily permease	–	–	0.97	75	+	
**P8F10**	**2380663**	**NCgl2167**	**Pyruvate dehydrogenase subunit E1**	**–**	**1.2.4.1**	**0.96**	**48**	**+**	**+**
**P8F12**	**1142652**	**NCgl1051**	**Hypothetical protein**	**+**	**–**	**0.95**	**55**	**+**	**+**
**P8G3**	**1690962**	**NCgl1533**	**GTP cyclohydrolase II**	**+**	**3.5.4.25**	**0.94**	**40**	**+**	**+**
P21D2	3119587	intergenic		–	–	0.98	81	+	
P21E9	1525622	NCgl1392	Hypothetical protein	+	–	0.98	99	+	
P21G4	2867647	NCgl2603	Cell division protein	–	–	0.98	87	+	
P21G11	199693	NCgl0181	Glutamate synthase large subunit	+	1.4.1.13	0.98	119	+	

### Analysis of Single Mutants Involved in Glutamate Metabolism

**Figure 6:**
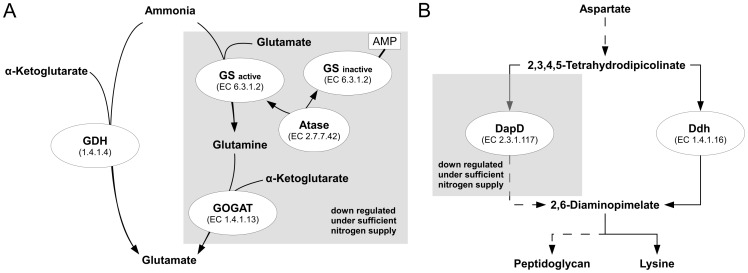
Schematic overview of the GS/GOGAT pathway (A) and the diaminopimelate biosynthesis (B). GDH: glutamate dehydrogenase; GS: glutamate-ammonia ligase; GOGAT: glutamate synthase; Atase: glutamate-ammonia ligase adenylyltransferase; DapD: tetrahydrodipicolinate succinylase; Ddh: diaminopimelate dehydrogenase. Dashed arrows represent more than one reaction.

**Figure 7:**
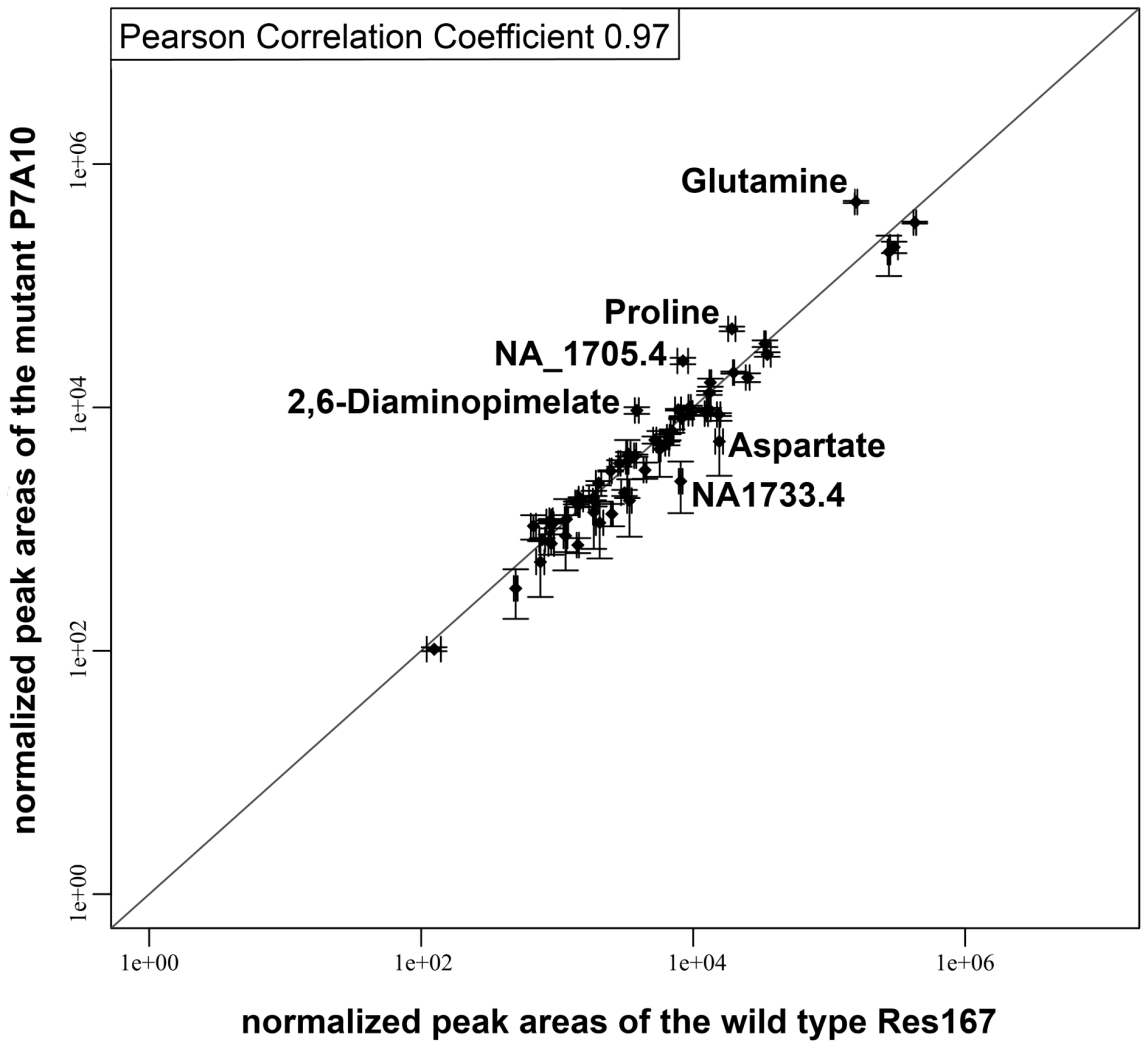
Metabolic profile of the C. glutamicum mutant P7A10. Scatter plot of normalized peak areas of the mutant P7A10 against the wild type Res167. Axes are logarithmically plotted. Only metabolites with changes >2 are labeled.

The detailed analysis of the metabolome of 258 mutants would go beyond the scope of this study. Therefore, we focused the analysis on single mutants that were linked to the glutamate metabolism, either on the genomic or the metabolic level. The mutant P7A10 takes a special position among the analyzed mutants. Not only the function of the disrupted gene is known, but also the metabolic profile of a deletion mutant of the same gene has been already analyzed and published by Rehm et al. [31]. The affected gene is *glnE* (*NCgl2147*), encoding an adenylyltransferase (EC 2.7.7.42), which is part of the ammonium assimilation in *C. glutamicum* and catalyzes the adenylation/deadenylation of the glutamate-ammonia ligase (EC 6.3.1.2). The organism possesses two ways to assimilate ammonium (figure 6A), the glutamate dehydrogenase pathway (GDH) and the glutamine synthetase/glutamate synthase pathway (GS/GOGAT). While the glutamate dehydrogenase pathway is permanently active and has only a low affinity to its substrates, the GS/GOGAT system is regulated by the supply of nitrogen and shows a high affinity to ammonium. As the GS/GOGAT pathway consumes one extra mole of ATP per mole of fixed ammonium compared to the GDH pathway [32], it is down-regulated when nitrogen is available in sufficient amounts. One mechanism of regulation of the GS/GOGAT system is the inactivation of the glutamate-ammonia ligase (EC 6.3.1.2, formerly known as glutamine synthetase) by adenylation, caused by adenylyltransferase. A mutation of the adenylyltransferase therefore leads to an increased activity of glutamate-ammonia ligase [33] and the pathway remains active, even under sufficient nitrogen supply. Rehm et al. [31] reported that deletion of the gene *glnE* (*NCgl2147*, encoding the adenylyltransferase) results in a decreased glutamate (1.5 fold) and a highly increased glutamine concentration (5 fold). The analysis of these metabolites was based on determination of absolute metabolite concentrations using a LC/MS setup and the tendencies could be reproduced by our GC/MS analysis. In our analysis the glutamate-ammonia ligase substrate glutamate was 0.69 fold lower while the product glutamine was 3.1 fold higher concentrated in the mutant (a table with all metabolite ratios for every mutant is shown in table S3). Additionally, the substrate of the GS/GOGAT system, α-ketoglutarate, showed a 0.69 fold decrease. Many other observations, made by Rehm et al. [31] concerning metabolites of the central metabolism were confirmed proving the comparability of our transposon mutant and the described deletion mutant. We observed some differences, that were not mentioned by Rehm et al. [31]. A scatter plot in figure 7 shows an overview over the strongest changes of the metabolic profile of the mutant P7A10 compared to the wild type. In addition to the increased amount of glutamine (3.1 fold) the mutant showed higher concentrations of proline (2.3 fold), an unidentified metabolite NA_1705.4 (2.9 fold) and 2,6-diaminopimelate (2.5 fold). Furthermore, decreased levels were determined for aspartate (0.34 fold) and the unidentified metabolite NA_1733.4 (0.6 fold).

Similar to the mechanism of ammonium assimilation, *C. glutamicum* possesses two ways for the biosynthesis of 2,6-diaminopimelate [34] (figure6 B). One pathway involves the diaminopimelate dehydrogenase (Ddh, EC 1.4.1.16), which shows only a low affinity for ammonium, but is permanently active. The second pathway involves a tetrahydrodipicolinate succinylase (DapD, EC 2.3.1.117), which has a high affinity for its substrate and is regulated by AmtR [35], the master regulator of nitrogen control in *C. glutamicum*. The increased level of 2,6-diaminopimelate in the mutant together with the decreased aspartate level indicate a connection between the regulation of the GS/GOGAT system and the regulation of the 2,6-diaminopimelate synthesis. It seems that an inactivation of the glutamate-ammonia ligase adenylyltransferase results in an increased synthesis of 2,6-diaminopimelate, which might originate from an increased flux through the nitrogen dependent pathway involving the tetrahydrodipicolinate succinylase.

The increased amount of glutamine in the mutant P7A10 could be traced back to the GS/GOGAT system. Together with the increased glutamine concentration the mutant showed an increased level of proline. A similar, but even stronger response for these metabolites has been detected for the mutant P7E1 (figure 5B). For this mutant glutamine showed an 8 fold and proline a 12 fold increased level compared to the wild type. A connection between these two metabolites was shown during the response to osmotic stress. Wolf et al. [36] showed that proline and glutamine are the preferred compatible solutes in *C. glutamicum* formed under sufficient nitrogen supply. While glutamine is produced as a fast reaction to osmotic stress, proline is the slightly delayed main reaction of the organism and its concentration in the cell increases about eightfold upon growth at high osmolarity. Since an osmotic shock during cultivation of our strains can be excluded, the accumulation of glutamine and proline in the mutants must be explained by the mutations caused by the insertion of the transposon. For the mutant P7E1, the insertion point of the transposon could not be identified which makes an interpretation impossible. But for P7A10 the increased proline level might be induced by the high activity of the glutamate-ammonia ligase caused by the mutation in *glnE* (*NCgl2147*) encoding the corresponding adenylyltransferase (EC 2.7.7.42).

Another mutant which showed an increased proline level is P8F12. The function of the disrupted gene *NCgl1051* is still unknown. As shown in figure 5B, this mutant had a highly increased proline level (10 fold), but only a slightly increased level of glutamine (1.4 fold). The level of glutamate in P8F12 was increased 1.5 fold compared to the wild type, while the N-acetyl-glutamate level was strongly reduced in the mutant (0.07 fold). In addition to the glutamate associated metabolites, the TCA cycle was also strongly affected in this mutant (figure 5A). While citrate was slightly increased (1.3 fold), the later TCA cycle metabolites showed decreased concentrations in the mutant. The decrease of metabolite levels becomes stronger from α-ketoglutarate (0.61 fold) and succinate (0.60 fold) over fumarate (0.36 fold) up to malate (0.33 fold). These strong changes also affect the growth of P8F12, which is reduced to 55% compared to wild type after growth on glucose minimal medium for 7 hours. For the highly decreased concentration of N-acetyl-glutamate detected in this mutant, two reasons are possible: One reason would be an indirect effect of the highly increased proline concentration. As Lee et al. [37] reported, proline reduces the binding of the transcriptional regulator ArgR upstream of *argB* (coding for the acetylglutamate kinase, EC 2.7.2.8). This results in an increased activity of the acetylglutamate kinase and, in consequence, its substrate N-acetyl-glutamate is decreased. A second possible reason for the highly decreased N-acetyl-glutamate concentration is a function of the affected gene *NCgl1051* in the arginine biosynthesis. Although the function of this gene is still unknown, Silberbach et al. [38] showed that under ammonium limitation the transcription of this gene is reduced by 0.5 compared to normal growth conditions. Besides other genes, all genes coding for the arginine biosynthesis (*argBCDFGHJRS*) showed reduced transcription under ammonium limitation in this study. The described reduced transcription under ammonium limitation together with the highly reduced N-acetyl-glutamate concentration in the transposon mutant might indicate a function of the gene in the arginine biosynthesis. Unfortunately, the concentration of arginine and ornithine were under the detection limit and could not be measured in our study, so that a proof of this hypothesis is currently missing.

Another gene that showed a transciptional response to ammonium limitation in the study of Silberbach et al. [38] is *gltB* (*NCgl0181*), which is affected in the mutant P21G11. On opposite to the gene *NCgl1051* (P8F12), the transcription of *gltB* is enhanced by a factor of 10 compared to normal growth conditions. The gene *gltB* (*NCgl0181*) is coding for the glutamine 2-oxoglutarate aminotransferase large subunit (EC 1.4.1.13). Together with the small subunit GltD, the two proteins form the GOGAT part of the GS/GOGAT system in *C. glutamicum* and catalyze the synthesis of glutamate from α-ketoglutarate and glutamine (figure 6A). Besides the mutant P7A10 (*NCgl2147*), this is the second mutant with a mutation in the GS/GOGAT system. As explained before, the GOGAT part is not essential for *C. glutamicum* under high nitrogen supply. Beckers et al. [39] showed that the expression of the operon formed by *gltB* (*NCgl0181*) and *gltD* (*NCgl0182*) is under control of the repressor protein AmtR. In consequence, a mutation should have no effect under sufficient nitrogen supply. Indeed the transposon mutant showed no changes in the glutamate associated metabolites and did not appear in the screening (figure 5B). Still the mutant showed minor changes in the concentration of the TCA cycle intermediates (figure 5A) and increased levels for serine (2.3 fold) and glycine (2.2 fold), which indicates that the mutation is not silent.

## Discussion

As shown by Choorapoikayil et al. [40], the analysis of metabolite levels permits to clarify predicted gene functions. To analyze not only single, but many mutants in parallel, we established a method for the high-throughput analysis of a transposon mutant library of *C. glutamicum*. We divided the method into two parts: first, investigation of the genomic background and, second, the analysis of the metabolic profiles of the mutants.

We were able to identify the accurate position of the transposon in 722 mutants within a short time. Beside the identification of disrupted genes in mutants, we were interested in the distribution of the insertion sites in the genome. We could confirm the random integration of the vector pAT6100 into the genome of *C. glutamicum*, without any site preference (figure 1). A review of the insertion points revealed that for more than a third of the genes of the identified mutants no or only a hypothetical function could be assigned by bioinformatic methods.

Analysis of the growth distribution of 258 randomly chosen mutants showed, that the analyzed mutants can be divided into four groups, from no growth (<40%) over reduced growth (40–80%) up to similar (80–120%) and even enhanced growth (>120%) compared to the wild type on glucose minimal medium. The majority of the mutants (80%) showed a comparable growth to the wild type. The growth behavior of the mutants together with the identified insertion points provide important information suitable for the detailed understanding of *C. glutamicum*. Only 22 of the 258 investigated mutants were not able to grow on minimal medium, whereas five mutants showed an increased growth compared to the wild type. For a detailed discussion of the growth of single mutants further experiments need to be done. Nevertheless, our current findings demonstrate the possibilities, which a transposon mutant library with identified mutations presents.

We applied our in-house developed software MetaboliteDetector to process the huge amount of GC/MS data. With the aid of this tool we obtained a very low overall relative error of 11.5% for the metabolome samples of the quality standard. In total, almost 300 substances with biological response were detected, which is a very good coverage for the detailed analysis of the metabolism.

A pairwise correlation of the metabolites of all analyzed mutants was performed to get a cross section of the dependencies of the metabolites in *C. glutamicum* (figure 3). These correlations reflect many known dependencies like the correlation of the metabolites of the TCA cycle, the correlation of the fatty acids or the correlation of the amino acids glycine and serine. The reduction of the complexity in a dendrogram (figure 4) showed interesting clusters of the metabolites. Besides already known dependencies of metabolites, this view enabled the allocation of so far non identified metabolites to groups of known metabolites. This provides the opportunity to gather further information about the identity of these metabolites. Since a metabolite interacts with many other metabolites, the complexity of the metabolism is very high. Additionally, only parts of the metabolic pathways can be investigated with the present analytical methods as the metabolite classes are chemically too diverse to be detected by one method. But still, for limited parts of the metabolism like the TCA cycle, dependencies are well represented by the pairwise correlations.

To get a first inside into the large amount of data we filtered the data for interesting mutants by searching for specific patterns of closely related metabolites, as shown in figure 5. For the TCA cycle, changes in the metabolite level reflect the position of the metabolites in the pathway relatively well. While citrate and α-ketoglutarate seem to be regulated independently of the other intermediates, succinate, fumarate and malate are mostly co-regulated. Further, fumarate and malate often share the same fold change which underlines the close connection of the two metabolites.

Compared to the TCA cycle, the picture drawn by the ratios of glutamate associated metabolites in figure 5B is more complex. Because the metabolites are not part of a linear pathway, changes do not necessarily affect the other metabolites. But still, there are visible patterns and connections between mutants, as for P7E1 and P7A10, both showing an increased concentration of glutamine and proline.

The mutant P7A10 which is mutated in the gene *glnE* (*NCgl2147*) encoding the glutamate-ammonia-ligase adenylyltransferase (EC 2.7.7.42) offers a good opportunity to prove the high-throughput metabolic profiling method, as well as the phenotype of the mutant, as Rehm et al. [31] already analyzed a *glnE* deletion mutant in detail. A comparison of our results with the published results showed a high degree of accordance in the measured metabolite levels, which confirms the stability of the transposon and proves the comparable phenotypes resulting from a transposon mutant and a deletion mutant of the same gene.

Additionally, increased levels of 2,6-diaminopimelate and decreased levels of aspartate in the *glnE* mutant indicate a connection of the 2,6-diaminopimelate synthesis to the regulation of the GS/GOGAT system. As the 2,6-diaminopimelate production involving tetrahydrodipicolinate succinylase (DapD, EC 2.3.1.117) is regulated dependent on the nitrogen supply (figure 6B), it seems that the inactivation of the glutamate-ammonia ligase adenylyltransferase or the resulting increased activity of the glutamate-ammonia ligase are inducing an increased flux through the tetrahydrodipicolinate succinylase involving synthesis of 2,6-diaminopimelate in *C. glutamicum*.

The increased level of proline and glutamine in the mutant P7A10 were found with an even stronger response in the so far not identified mutant P7E1 (figure 5B). Glutamine and proline are relatively closely related in the metabolism and together they are part of the response to osmotic stress in *C. glutamicum* [36]. Since the regulation of the proline synthesis via *proA*, *proB, proC* is still unknown [41] we suspect that in the mutant P7A10 the inactivation of the glutamate-ammonia ligase adenylyltransferase or the resulting increased activity of the glutamate-ammonia ligase induces the synthesis of proline.

Altogether, the observations made for the mutant P7A10 indicate a central role of the glutamate-ammonia ligase adenylyltransferase in several nitrogen dependent reactions in *C. glutamicum*, which is supported by the speculations made by Rehm et al. [31] that this enzyme possesses a moonlighting function.

Raamsdonk et al. [42] have shown that it is possible to reveal gene functions by comparing metabolic responses of mutations in unknown genes with the response of mutations in genes with known function. The mutants P7A10 and P7E1 showed a high degree of accordance in their metabolic profiles, which indicates a possible gene function for the mutant P7E1 in the GS/GOGAT system as well. As the insertion point in this mutant could not be determined so far, any gene function prediction at this point is impossible and further analysis have to be done.

Another mutant, which showed an increased amount of proline (10 fold), is P8F12 (*NCgl1051*). For this mutant, a strongly decreased N-acetyl-glutamate pool (0.07 fold) was detected. For these extreme values two explanations are possible. At first, according to Lee et al. [37] proline reduces the binding of the ArgR regulator upstream of *argB*, which is encoding for the N-acetylglutamate kinase (EC 2.7.2.8). In consequence, the transcription of this enzyme is enhanced, which leads to a reduced intracellular concentration of its substrate N-acetyl-glutamate. The second explanation implies that the affected gene is involved in arginine biosynthesis. This hypothesis is supported by the reduced transcription of the gene *NCgl1051* under nitrogen limitation, which was found by Silberbach et al. [38] and correlates with the reduced transcription of the genes responsible for arginine biosynthesis. Contrary to the first explanation, a similar high concentration of proline in the mutant P7E1 showed only a slightly decreased N-acetyl-glutamate concentration (0.72 fold).

The mutation of the gene *gltB* (*NCgl0181)*, encoding for the glutamine 2-oxoglutarate aminotransferase large subunit (EC 1.4.1.13) in the mutant P21G11 affirms the subordinated role of the GOGAT part of the GS/GOGAT system during sufficient nitrogen supply. The mutant showed no significant changes in the glutamate associated metabolites. Furthermore the mutant showed good growth (119%) and a high correlation coefficient to the wild type (0.98). Although this indicates only a minor influence of the mutation, for a small number of metabolites significant changes were observed. These changes affect the TCA cycle intermediates succinate (1.4 fold), malate (1.7 fold) and fumarate (1.4 fold), as well as serine (2.4 fold) and glycine (2.0 fold), which all showed increased concentration compared to the wild type. The increased growth together with the increased concentration of the TCA cycle intermediates indicate an increased energy metabolism in the mutant. Based on the findings made by Beckers et al. [43] the gene *gltB* (*NCgl0181)* was expected to be repressed by AmtR during sufficient nitrogen supply. Therefore, the origin of the observed changes in the metabolism as a response to the introduced transposon in this gene remains to be elucidated.

## Conclusion

Taken together the described combination of high-throughput transposon insertion site determination and metabolome analysis allows to investigate many gene functions at once. The here described first analysis of selected mutants shows the confirmation of already known gene functions and indicates potential functions for genes with unknown function. In many cases metabolic profiling alone is not able to prove a gene function and therefore, additional information has to be gathered. The integration of systems biology data from different levels would greatly complement the collected data and together this would build up a highly promising source of knowledge about microorganisms.

## Materials and Methods

### Strains, Media and Cultivation

The restriction-deficient wild type derivative strain Res167 of *Corynebacterium glutamicum* ATCC 13032 was used for the creation of the transposon mutant library. It was also used in metabolic measurements as the wild type reference. Transposon mutants of *C. glutamicum* were generated with the IS*6100*-based pAT6100 artificial transposon vector similar to the published method of Mormann et al. [13]. The construction of the vector was described by Tauch et al. [21]. Media composition and cultivation procedure were already explained in detail by Börner et al. [3]. According to that, cultivation for metabolome analysis were done in minimal medium (MM1): One liter of MM1 contained 5 g (NH_4_)_2_SO_4_, 5 g urea, 2 g K_2_HPO_4_*3H_2_O, 2 g KH_2_PO_4_, 0.25 g MgSO_4_*7H_2_O, 0.01 g CaCl_2_, 0.2 mg biotin, 20 g glucose, 28.5 mg FeSO_4_*7H_2_O, 16.5 mg MnSO_4_*H_2_O, 6.4 mg ZnSO_4_*7H_2_O, 0.764 mg CuSO_4_*5H_2_O, 0.128 mg CoCl_2_*6H_2_O, 0.044 mg NiCl_2_*6H_2_O, 0.064 mg Na_2_MO_4_*2H_2_O, 0.048 mg H_3_BO_3_, 0.05 mg SrCl_2_, 0.05 mg BaCl_2_, 0.028 mg KAl(SO_4_)_2_*12H_2_O.

### Determination of the Transposon Insertion Sites

The genomic DNA of transposon mutants was extracted with the Nucleospin96-Kit by Macherey-Nagel (Macherey-Nagel GmbH, Düren, Germany) following the manufactures instructions. DNA concentration and purity were measured with the Tecan InfiniteM200-Photometer (Tecan Group Ltd., Männedorf, Switzerland) combined with a NanoQuant Plate. The thermal asymmetric interlaced PCR (TAIL) was done as described by Liu et al. [15]. Three transposon-specific primers for each border (first border: LR01: AGTGATCTGCACCAATCTCGACTAT; LR02: GGAAAGCTCAAGATACTGATCAAGC; LR03: GTGGAGAGAGCTTTTGGCATTG; second border: LR04: GTTTTGTCGCGTATGTCCTAAGTTGT; LR05: CTGATCGGATAGCGACAATACCAG; LR06: CATGCTCAAGCTTCACGATTTTTG) and two degenerated primers (AD2: TGWGNAGSANCASAGA and AD5: NTCGASTWTSGWGTT) were designed. TAIL PCR was carried out twice for a border, once with each degenerated primer. The amplification products of the third reaction were analyzed by gel electrophoresis. Adequate amplification products were selected for purification and sequencing done by GATC Biotech (GATC Biotech AG, Konstanz, Germany). For sequencing the third TAIL-primer (LR03/LR06) was used. The accurate insertion point of the transposon was determined by using the resulting sequences in a BLAST search against the *C. glutamicum* genome (NCBI).

### Sample Preparation and Measurement

The sample preparation for metabolome analysis was described by Börner et al. [3]. Only minor changes have been made: the Ethanol-Ribitol solution was substituted with a Methanol-Ribitol solution. Determination of the optical density was performed in triplicates. A quality standard was generated by pooling 200 wild type extracts, portioning and drying them afterwards. These samples were stored at −80°C. Six quality standard samples were distributed over each experiment. For derivatization and measurement the Leco Pegasus 4D GCxGC TOFMS (Leco Instrumente, Mönchengladbach, Germany) combined with an Agilent 7890A (Agilent Technologies, Böblingen, Germany) gas chromatograph and a Gerstel MPS 2 XL Twister (Gerstel, Mühlheim a. d. Ruhr, Germany) autosampler was used. For on-line derivatization the dried samples were redissolved and derivatized with 40 µl pyridine, containing 20 mg · ml^−1^ methoxyamine hydrochloride, at 30°C for 90 min under shaking. After adding 60 µl MSTFA (N-methyl-N-trimethylsilyltrifluoroacetamide), samples were incubated at 37°C for 30 min under shaking, followed by incubation at room temperature for 90 min. A retention index marker (n-alcanes ranging from C10…C36 in cyclohexane) was used to convert retention times to retention indices. GC/MS analysis was performed on a Leco Pegasus 4D GCxGC TOF mass spectrometer in the one dimensional mode. In summary, 1 µl was injected into a PTV (Gerstel, Mühlheim a. d. Ruhr, Germany) splitless at 70°C. After initial time of 0.2 min the injector was ramped at 14°C s^−1^ to a final temperature of 280°C and held for 5 min. The Agilent 7890A gas chromatograph was equipped with a VF-5MS column (30 m×0.25 mm I.D.) (Varian Deutschland, Darmstadt, Germany). The GC was operated at constant flow of 1 ml · min^−1^ helium. The temperature program started at 70°C held for 1 min, followed by temperature ramping of 10°C min^−1^ to a final temperature of 350°C, which was held constant for 6 min. The transfer line temperature was set to 300°C. Ion source temperature was adjusted to 250°C. Full-scan mass spectra of m/z 45…600 were collected at an acquisition rate of 8 scans sec^−1^. Solvent delay time was 5 min. For data acquisition ChromaTOF, version 4.24 (Leco Corporation) was used.

### High-throughput Data Processing and Compound Identification by MetaboliteDetector

For the processing of GC/MS data we used the version 2.0.7 of our in-house developed software MetaboliteDetector [17]. The peak identification was performed in a non-targeted mode with a combined compound library. This library was achieved by a merge of our in-house library with the Golm metabolome database [28] and comprises about 2500 compounds. After processing, non biological and artificial peaks were eliminated by the aid of blanks. Peak areas were normalized by optical density of the sample and the internal standard (ribitol). Peak areas of derivatives were summarized to metabolites. Metabolites with a reproducibility of under 80% within an experiment were discarded for further analysis. For compatibility of peak areas from the single experiments, metabolites were first normalized by a metabolite specific, then by a sample specific median.

### Statistical Analysis

For the reference, consisting of 45 wild type samples, as well as for every mutant, consisting of three biological samples, and the quality standards, consisting of six samples per experiment, the mean, median and the relative standard error of each metabolite was determined. Furthermore, for each metabolite a ratio of the peak area of the mutant to the peak area of the wild type reference was computed by using mean values. The Pearson correlation coefficient of the logarithmized peak areas was calculated. Significance of changes in metabolite level were computed by a Student’s T-Test with Bonferroni corrected p-value for multiple testing based on a p-value of 0.01. For the pairwise correlation analysis of metabolites, the ratios between the median peak area of every mutant and the median peak area of the wild type were calculated and afterwards log2 transformed. Only highly reproducibly quantified metabolites (>95% over all samples) were used, to perform a pairwise correlation after Pearson of all metabolites over all mutants.

## Supporting Information

Table S1 **List of all identified insertion points of** ***C. glutamicum*** **transposon mutants that have been investigated by metabolic profiling.** The table shows the determined position of the transposon as well as the corresponding gene locus and gene description. Additionally, for each mutant, the correlation to the wild type and the relative growth compared to the wild type are shown.
(XLS)

Table S2 **List of all reproducibly quantified metabolites in the wild type showing the relative standard error and the signal to noise ratio for each metabolite.**
(XLS)

Table S3 **Table of mutant to wild type ratios of the mean peak areas for every metabolite and all mutants.**
(XLS)
